# Eating Disorders: Prevalence of Bulimia Nervosa Among Tunisian Military Nursing Students

**DOI:** 10.1192/j.eurpsy.2025.1391

**Published:** 2025-08-26

**Authors:** Y. Ben Othmene, S. Bejaoui, W. Kabtni, A. Baatout, S. Eddif, I. Gafsi, H. Kefi, A. Oumaya

**Affiliations:** 1 Psychiatry, Military Hospital of Instruction, Tunis, Tunisia

## Abstract

**Introduction:**

Eating disorders are life-threatening. They affect both physical and mental well-being, specially among young adults in high-stress environments such as military training.

Bulimia Nervosa (BN) is characterized by binge eating and inappropriate compensatory behavior to control weight with potentially dangerous sequelae.

Early recognition is essential to optimise treatment management.

**Objectives:**

This study sought to determine the prevalence of Bulimia Nervosa, its symptoms and its severity, among military nursing students in Tunisia.

**Methods:**

Between March and May of 2024, **a** cross-sectional descriptive survey was conducted for nursing students at the Tunisian Military Health School. Data were gathered using a data file and a self-report questionnaire: Bulimic Investigatory Test, Edinburgh (BITE). The BITE is designed to identify binge eating and compensatory behaviors, providing information on cognitive and behavioral aspects of bulimia nervosa. The scores are classified into two subscales: symptoms and severity. Excel software was used to analyze the obtained data.

**Results:**

The study enrolled 148 nursing students with 57.4% male. The average age was 21.3 [19-24] years. Among our participants, 57.4% came from northern Tunisia. Of them, 48.6% were in their third year and 19.6% in their second one. Of the surveyed, 85.1% were in a middling socioeconomic position followed by 9.5% in a high one.

With an average weight of 75.1±14.8 [48-105]kg, 47.3% weighed more than 70kg with 4.05% between 90kg and 100kg and 2.02% exceeding 100kg. Of the students, 67.56% were taller than 1m70. The average Body Mass Index BMI was 23[17-32]. Around 71.6% had a normal BMI, while 24.32% were overweight[BMI:25-30], and 1.35% were obese[BMI>=30].

In terms of physical appearance, 89.9% expressed satisfaction with their bodies’ looks and 67.6% with their weight. Growing up, 73.6%(N=109) did not have any weight issues while 8.1%(N=12) reported they were obese.

Prior to completing the BITE, 57.4% said they had no eating disorders while 18.2% estimated they had BN.

Of the surveyed, 83.3% expressed dissatisfaction with school meals mainly due to insufficient quantity, poor quality, or tasteless food.

According to the study, the BITE’s average score was 8±6 [0-34]. Of the participants, 17.5%(N=26) had Bulimia Nervosa with a BITE score of 20 or above. The majority (76.92%,N=20) were female with one reported severe case; their average BITE’s score was 23±15 [1-32]; the male’s BITE average score was 20±9 [0-34].

Furthermore, according to the survey results, students suffering from BN had a BMI of 29±5 [weight:81±11kg] whereas those without eating disorders had a BMI of 25±3 [weight:69±12kg].

**Conclusions:**

This study highlights the remarkable prevalence of Bulimia Nervosa among Tunisian military nursing students underscoring the urge to pay attention to early symptoms for better targeted interventions and mental health promotion.

**Disclosure of Interest:**

None Declared

